# A Baffling Bump: A Case Report of an Unusual Chest Wall Mass in a Pediatric Patient

**DOI:** 10.5811/cpcem.2021.3.51958

**Published:** 2021-07-27

**Authors:** Haley Vertelney, Margaret Lin-Martore

**Affiliations:** University of California, San Francisco, Department of Emergency Medicine, San Francisco, California

**Keywords:** Necrotizing pneumonia, empyema necessitans, infectious disease, ultrasound, pediatric, case report

## Abstract

**Introduction:**

Chest wall masses are rare in children, but the differential diagnosis is broad and can include traumatic injury, neoplasm, and inflammatory or infectious causes. We report a novel case of an eight-year-old, previously healthy female who presented to the emergency department (ED) with one month of cough, fevers, weight loss, and an anterior chest wall mass.

**Case Report:**

The patient’s ultimate diagnosis was necrotizing pneumonia with pneumatocele extending into the chest wall. This case is notable for the severity of the patient’s pulmonary disease given its extension through the chest wall, and for the unique speciation of her infection.

**Conclusion:**

Although necrotizing pneumonia is a rare complication of community-acquired pneumonia, it is important for the emergency physician to recognize it promptly as it indicates severe progression of pulmonary disease even in children with normal and stable vital signs, as in this case. The emergency physician should consider complications of pneumonia including pneumatocele and empyema necessitans when presented with an anterior chest wall mass in a pediatric patient. Additionally, point-of-care ultrasound was used in the ED to facilitate the diagnosis of this illness and was particularly useful in determining the continuity of the patient’s lung infection with her extrathoracic chest wall mass.

## INTRODUCTION

The emergency physician must consider a wide differential diagnosis for a pediatric patient with an acquired chest wall mass. An incomplete list of some of the most common etiologies includes trauma, neoplasm (most commonly lymphoma, germ cell or neurogenic tumors, sarcoma, lipoma), and inflammatory or infectious causes (abscess, granuloma, osteomyelitis, cellulitis).^4^ We present a case of an anterior chest wall mass in an eight-year-old, previously healthy female patient that was ultimately determined to be an extrathoracic extension of a necrotizing pneumonia of the right lung. We found no other reports in the literature describing a previously healthy pediatric patient with a chest wall mass arising from a necrotizing pneumonia communicating through the chest wall. We present this case to encourage the consideration of necrotizing pneumonia in a pediatric patient with a chest wall mass.

## CASE REPORT

An eight-year-old, previously healthy female presented to the emergency department (ED) with one month of weight loss, fatigue, dry cough, and low-grade fevers. She had been seen one month prior to presentation for cough and fatigue at a clinic and was given return precautions for a presumed viral upper respiratory infection. Over the course of the subsequent month, her symptoms worsened to include weight loss, fatigue, and persistent low-grade fevers. She presented again to the clinic and was found to have labs significant for hemoglobin of 6.5 grams per deciliter (g/dL) (reference range: 11.6–15.5 g/dL), hematocrit of 19% (35.0–45.0%), white blood cell count of 12.5×10^9^/ liter (L) (4.5–15.5 ×10^9^/L), and platelets of 544 ×10^9^/L (140–450 ×10^9^/L). The patient was then referred to the ED for further evaluation.

Initial vital signs included temperature of 36.9°C, heart rate of 145 beats per minute, blood pressure of 107/72 millimeters of mercury, respiratory rate of 34 breaths per minute, and oxygen saturation of 96% on room air at rest. Initial exam showed an ill-appearing, thin child in no apparent distress. She had multiple dental caries without tonsillar exudate or erythema, and no abscess noted around the teeth or in the soft tissues of the mouth, with midline uvula. Auscultation of the lungs revealed rhonchi with decreased air movement on the right. She had a 4-centimeter (cm) soft mass on her right anterior chest wall superior to the nipple in the mid-clavicular line without induration or erythema. Her abdomen was flat, soft, and nontender. Neurological examination was non-focal. Her skin was pale with scattered areas of hyperpigmentation on all extremities, and she had pale sclera.

The patient’s initial ED bloodwork was significant for a hemoglobin of 5.9 g/dL with hematocrit of 20.2%, white blood cell count of 9.4 ×10^9^/L (reference range: 4.5–15.5×10^9^/L), and platelet count of 412 ×10^9^/L (140–450×10^9^/L). C-reactive protein was 92.4 milligrams (mg)/L (mg/L) (0.1–1 mg/L). Her bloodwork was otherwise unremarkable including electrolytes within normal limits.

**Image 1:**
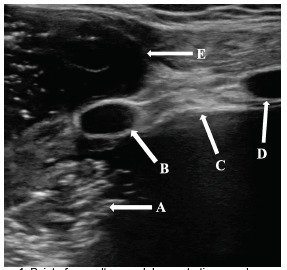
Point-of-care ultrasound demonstrating complex, extrathoracic, avascular heterogeneous fluid collection (E) that communicates with fluid with similar echogenic features in the patient’s intrathoracic space (A). C: Pleural line. B and D: Ribs.

Point-of-care ultrasound (POCUS) in the ED was performed to investigate the anterior chest wall mass using a linear transducer (Image 1). Heterogeneous hypoechoic fluid with scattered areas of echogenic air artifact was identified in the patient’s right chest. An extrathoracic small anterior chest wall collection demonstrated similar echogenic features with foci of gas and no evidence of internal vascularity. It appeared to communicate with the patient’s right lung.

**CPC-EM Capsule**

What do we already know about this clinical entity?

*Necrotizing pneumonia is a rare but serious complication of community-acquired pneumonia in children*.

What makes this presentation of disease reportable?

*We found no previously reported cases of necrotizing pneumonia in a pediatric patient that progressed through the chest wall to an extrathoracic pneumatocele*.

What is the major learning point?

*Community-acquired pneumonia can progress to necrotizing pneumonia and pleural infections involving the chest wall*.

How might this improve emergency medicine practice?

*Emergency physicians should consider advanced complications of pneumonia when presented with an acquired chest wall mass in a pediatric patient*.

**Image 2:**
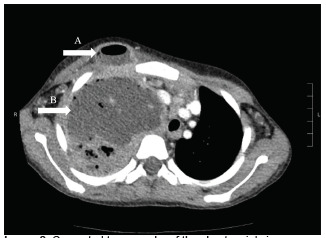
Computed tomography of the chest, axial view, demonstrating massive right hemithorax of multiloculated fluid (B) with extrathoracic fluid and air collection (A) at right anterior chest wall.

**Image 3:**
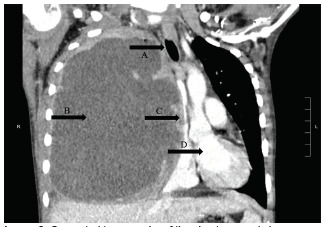
Computed tomography of the chest, coronal view. Massive hemithorax measuring 12.7 × 7.7 × 13.8 centimeters, (B) with leftward mediastinal shift (D) and occlusion of the right mainstem bronchus (A) with significant mass effect onto a patent superior vena cava (C).

Computed tomography of the chest (Images 2 and 3) showed massive right hemithorax 12.7 × 7.7 × 13.8 cm multiloculated fluid- and air-containing collection with an extrathoracic, rim-enhancing fistulous extension to the right anterior chest wall, measuring 3.3 × 2.0 cm. There was evidence of leftward mediastinal shift and occlusion of the right mainstem bronchus with significant mass effect onto a patent superior vena cava, with severe narrowing of the right main pulmonary artery and venous branches with arterial branches visualized distally.

The patient remained hemodynamically stable throughout her time in the ED and maintained adequate oxygenation with two liters of nasal cannula placed for comfort. Pediatric hematology/oncology was consulted from the ED for concern for possible malignancy. The patient was transfused three units of 5 milliliters (mL) per kilogram packed red blood cells and transferred to the pediatric intensive care unit (PICU).

The patient’s hospital course is summarized as follows: The right chest was aspirated, with approximately 400 mL of purulent material and blood drained in the operating room. A chest tube was placed to suction. The pleural cultures grew *Streptococcus pneumoniae*, *Staphylococcus aureus*, *Fusobacterium nucleatum*, and *Streptococcus viridans*. The patient completed a course of vancomycin and ampicillin and sulbactam before transitioning to amoxicillin clavulanate for three weeks of total antibiotic therapy. Pleural fluid cultures and imaging studies were as described previously. The patient intermittently required up to 10 L of high-flow nasal cannula during her PICU stay and elevated head of bed due to desaturations with activity. She was moved from the PICU on hospital day six to the floor on room air and was discharged to home after 19 total days hospitalized with a Heimlich valve in place with regular dressing changes and follow-up.

## DISCUSSION

Necrotizing pneumonia is a severe complication of community-acquired pneumonia (CAP) in children. It is characterized by a progressive bacterial pneumonia, often in a previously healthy and young (commonly less than five years of age) host. 5 Although it is relatively uncommon and occurs in less than 4% of children with CAP by some estimates,^5^,^6^ serious complications such as septic shock, respiratory failure, pyopneumothorax, and empyema have been reported.^1^,^2^ The most common pathogens are Streptococcus pneumoniae and Staphylococcus aureus. 5 Computed tomography (CT) is the most sensitive diagnostic tool, with blood and pleural fluid cultures supporting speciation. 5 The mechanism underlying the progression of bacterial pneumonia to necrotizing pneumonia is poorly understood but likely relates to both host susceptibility and bacterial virulence factors.^5^ Necrotizing pneumonia is often resistant to adequate antibiotic therapy and may require surgical treatment or drainage of pleural fluid and gas that cause mass effect in the chest.^2^,^5^,^7^ Despite its severity, long-term sequelae and death are uncommon, and patients typically achieve recovery 5–6 months following diagnosis.^5^

In contrast, empyema necessitans is a rare complication of pleural space infections and occurs when the infected fluid dissects from the pleural space spontaneously into the chest wall. These cases most often result from inadequate treatment of an empyema but can occur after a necrotizing pneumonia or pulmonary abscess.^8^,^9^ Actinomyces and S pneumoniae are the most common pathogens, and less typically can include S aureus, Streptococcus milleri, Fusobacterium nucleatum, Mycobacterium avium, Mycobacterium intracellulare, Burkholderia cepacia, and Nocardia asteroides.^10^

This case is noteworthy because the patient had microbiologic features of both necrotizing pneumonia as well as a pleural infection arising from several different bacterial species. Her pleural *viridans* streptococci and *Fusobacterium* were likely oral flora originating from her poor dentition and are more characteristic of a pleural infection. The extrathoracic fluid collection was initially postulated to be empyema necessitans arising from this pleural infection; however, subsequent imaging evidenced that her disease process was more consistent with a necrotizing right upper lobe pneumonia with a pneumatocele extending through the chest wall. Her imaging did not show enhancing thickened pleura or split pleural sign that would suggest empyema extending to empyema necessitans.

Secondly, we propose that POCUS was a key diagnostic component in the initial ED workup. This imaging modality demonstrated complex avascular heterogeneous hypoechoic fluid with scattered echogenic air artifact both in the patient’s lung and small anterior chest wall collection. The movement of the substance visible on POCUS suggested that the contents of the patient’s lung infection communicated with the extrathoracic fluid collection, a finding that was later corroborated by CT of the chest.

## CONCLUSION

We present a case of necrotizing pneumonia that is unique for its microbiologic profile as well as its severity and extension through the patient’s chest wall. Point-of-care ultrasound in the ED facilitated visualization of the communication between the patient’s pulmonary infection and the anterior chest mass, which was a key component in diagnosis of necrotizing pneumonia extending to the chest wall. Emergency physicians should consider necrotizing pneumonia in patients with persistent cough, fevers, fatigue, and weight loss and should consider extension of a pulmonary infection when presented with an acquired chest wall mass in a pediatric patient.
